# Cross-comparison of cardiac output trending accuracy of LiDCO, PiCCO, FloTrac and pulmonary artery catheters

**DOI:** 10.1186/cc9335

**Published:** 2010-11-23

**Authors:** Mehrnaz Hadian, Hyung Kook Kim, Donald A Severyn, Michael R Pinsky

**Affiliations:** 1 Department of Critical Care Medicine, University of Pittsburgh Medical Center, 230 Lothrop Street, Pittsburgh, PA 15261, USA; 2 Cardiothoracic Surgery, University of Pittsburgh Medical Center, 230 Lothrop Street, Pittsburgh, PA 15261, USA; 3 Current address: Eisenhower Medical Center, 39000 Bob Hope Drive, Rancho Mirage, CA 92270, USA

## Abstract

**Introduction:**

Although less invasive than pulmonary artery catheters (PACs), arterial pulse pressure analysis techniques for estimating cardiac output (CO) have not been simultaneously compared to PAC bolus thermodilution CO (COtd) or continuous CO (CCO) devices.

**Methods:**

We compared the accuracy, bias and trending ability of LiDCO™, PiCCO™ and FloTrac™ with PACs (COtd, CCO) to simultaneously track CO in a prospective observational study in 17 postoperative cardiac surgery patients for the first 4 hours following intensive care unit admission. Fifty-five paired simultaneous quadruple CO measurements were made before and after therapeutic interventions (volume, vasopressor/dilator, and inotrope).

**Results:**

Mean CO values for PAC, LiDCO, PiCCO and FloTrac were similar (5.6 ± 1.5, 5.4 ± 1.6, 5.4 ± 1.5 and 6.1 ± 1.9 L/min, respectively). The mean CO bias by each paired method was -0.18 (PAC-LiDCO), 0.24 (PAC-PiCCO), -0.43 (PAC-FloTrac), 0.06 (LiDCO-PiCCO), -0.63 (LiDCO-FloTrac) and -0.67 L/min (PiCCO-FloTrac), with limits of agreement (1.96 standard deviation, 95% confidence interval) of ± 1.56, ± 2.22, ± 3.37, ± 2.03, ± 2.97 and ± 3.44 L/min, respectively. The instantaneous directional changes between any paired CO measurements displayed 74% (PAC-LiDCO), 72% (PAC-PiCCO), 59% (PAC-FloTrac), 70% (LiDCO-PiCCO), 71% (LiDCO-FloTrac) and 63% (PiCCO-FloTrac) concordance, but poor correlation (*r*^2 ^= 0.36, 0.11, 0.08, 0.20, 0.23 and 0.11, respectively). For mean CO < 5 L/min measured by each paired devices, the bias decreased slightly.

**Conclusions:**

Although PAC (CO_TD_/CCO), FloTrac, LiDCO and PiCCO display similar mean CO values, they often trend differently in response to therapy and show different interdevice agreement. In the clinically relevant low CO range (< 5 L/min), agreement improved slightly. Thus, utility and validation studies using only one CO device may potentially not be extrapolated to equivalency of using another similar device.

## Introduction

Although the pulmonary arterial catheter (PAC) measures cardiac output (CO) easily at the bedside in critically ill patients [1-3], the recent trend in intensive care unit (ICU) monitoring is toward minimally invasive methods [4-8]. Arterial pulse contour and pulse power analyses have emerged as less invasive alternatives to PAC-derived CO measures [9,10]. The accuracy of these devices for PAC-derived CO measures has not been systematically compared in response to therapies other than volume resuscitation [11,12]. These devices use different calibration schema and model the transfer of arterial pulse pressure to stroke volume differently. Thus, their cross-correlations may not be assumed to be similar. The LiDCO Plus™ (LiDCO Ltd, London, UK) uses a transthoracic lithium dilution estimate of CO for calibration, whereas the PiCCO Plus™ (Pulsion Ltd, Munich, Germany) uses a transthoracic thermodilution approach to compensate for interindividual differences in arterial compliance [13-15]. The FloTrac™ calculates CO from the pulse contour using a proprietary algorithm and patient-specific demographic data [16] with, however, inconsistent reports of accuracy [17-20].

Although all devices have been compared individually to PAC-derived estimates of CO, none have been compared to each other [21]. Oxygen delivery (DO_2_) targeted resuscitation algorithms may improve outcomes in selected patient groups [22]. Thus, knowing the degree to which different systems co-vary is important if one is to use these outcome studies in a general fashion to define the utility of all minimally invasive monitoring systems. Accordingly, in this study, we cross-compared the CO values and their changes in a critically ill patient cohort in whom active changes in blood volume, vasomotor tone and contractility were induced by specific therapies. We compared three pulse contour devices (LiDCO Plus, PiCCO Plus and FloTrac) (Edwards Lifesciences, Irvine, CA, USA) and two PAC thermodilution techniques: CO by thermodilution (COtd) and continuous cardiac output (CCO) in postoperative cardiac surgery patients during the first 4 postoperative ICU hours when most of the aggressive treatments occurred. To minimize initial CO differences, we calibrated the PiCCO and LiDCO devices using the initial PAC CO values, whereas the FloTrac did not allow external calibration.

## Materials and methods

The study was approved by our Institutional Review Board, and all patients provided signed informed consent. Twenty postcardiac surgery patients (age range, 54 to 82 yr) were studied. Additional inclusion criteria were the presence of both an arterial catheter and PAC (Edwards LifeSciences, Irvine, CA, USA) (either CO_TD _or CCO). Exclusion criteria were evidence of cardiac contractility dysfunction (ejection fraction < 45% by intraoperative echocardiography), pregnancy, having pacemaker or automated implantable cardioverter-defibrillator, persistent arrhythmias, heart and/or lung transplant, severe valvular (mitral, aortic, pulmonic or tricuspid) stenosis or insufficiency after surgery, intra-aortic balloon pump or other mechanical cardiac support.

Patients were admitted to the ICU on assist control ventilatory mode with 12/min respiratory rate (no patient had a spontaneous respiration > 16/min) and 6 ml/kg tidal volume, inspiratory-to-exporatory (I/E) time of 1:2 and 5 cm H_2_O positive end-expiratory pressure. Fentanyl (25-50 μg) was given as needed by nursing staff if the patient appeared to have pain or discomfort.

### FloTrac™ and PAC

The FloTrac™ pulse contour device (Vigileo™, Edwards LifeSciences, Irvine, CA, USA) was attached to the existing arterial cannula, and its sensor was attached to the processing or display unit to read CO. The patient's demographic data (height, weight, age, and gender) were entered into the device as recommended by the manufacturer. FloTrac CO is reported as an averaged value over 20 seconds using a proprietary algorithm [23]. All continuous CO measurements were collected from the Vigileo™ monitor and input into a WinDaq data acquisition system (WINDAQ V 1.26, Dataq Instruments Inc., Akron, OH) as previously described [24].

Either a CO_TD _or a CCO was measured by a standard PAC attached to Vigilance™ monitor (Edwards LifeSciences, Irvine, CA). If a non-CCO PAC was present, then CO measurements were taken upon patient arrival to the ICU and then after each therapeutic intervention as described below. CO_TD _was taken as the mean of at least three 10-ml 5°C 0.9 N NaCl bolus injections random to the respiratory cycle. The accuracy and acceptability of each thermal decay curve was judged visually on the attached ICU monitor. If CCO PAC was present, then all CCO data based on STAT values were continuously collected until end of the study using the WinDaq data acquisition.

### LiDCO plus™ and PiCCO plus™

Arterial wave form data was collected using the WinDaq data acquisition system as previously described [24]. These waveforms were then reinjected into both the LiDCO plus™ and PiCCO plus™ devices offline to calculate CO. To minimize differences due to initial calibration variance, both the LiDCO plus™ and PiCCO plus™ devices had their initial CO values taken from the simultaneous PAC-derived CO values at time 0 as recommended by the manufacturers, after which time neither device was recalibrated. All continuous LiDCO and PiCCO CO measurements were collected in a data acquisition system installed internally in the device. The clocks on the all data acquisition systems were matched. All the CO_TD _measurements were taken by one investigator (MH).

### Protocol

We compared the mean paired CO values 30 s before and 1-2 min after ending a volume challenge and after heart rate and blood pressure stabilized (< 5% variation over 30 s) following changes in vasoactive and inotropic therapy. We made no attempt to alter the usual care of the patients. The FloTrac data were blinded to the primary care physicians. All paired event data were downloaded in a common Microsoft Excel (Microsoft Corp., Redmond, WA, USA) spreadsheet for statistical analysis.

### Statistical analysis

We performed analysis of variance for comparison of mean baseline CO between the three devices. A *post hoc *Student's paired *t*-test was used to compare groups when significance was identified. *P *< 0.05 was considered significant. We performed Bland-Altman analysis for paired devices PAC-LiDCO, PAC-PiCCO, PAC-FloTrac, LiDCO-PiCCO, LiDCO-FloTrac and PiCCO-FloTrac. Bias was defined as the mean difference between CO measurements by each set of paired devices. The upper and lower limits of agreement were defined as ± 1.96 standard deviation (SD) of the bias. The percentage error was calculated as limits of agreement divided by the mean CO [25,26]. Bias, limits of agreement and percentage error were calculated for the entire data set for each set of paired devices and then separately for CO_TD _and CCO. We also performed two additional Bland-Altman analyses. We selectively compared limits of agreement and bias of CO values < 5L/min to ascertain whether any observed bias was selectively due to higher flow rates, which would have less clinical relevance. Since there is no reference CO measure, we also created a pooled CO measure as the mean of all the devices' CO values at one point (*Z*-statistic) and performed a Bland-Altman analysis of each device against this mean of all devices. For this analysis, we pooled the PAC COtd and CCO values into one variable. Since directional changes in CO are important in assessing response to therapy, the degree of concordance was defined as the percentage of the total number of events when paired devices showed the same directional change in CO (greater than ± 0.5 L/min) divided by the total number of events using a Pearson product-moment correlation coefficient analysis. We assumed that all paired CO data that varied by < 0.5 L/min reflected no change and then calculated the percentage of paired data points when both devices reported no change or a change of > 0.5 L/min in the same direction. We also calculated the correlation of the dynamic changes in these paired values using simple linear correlation analysis.

## Results

Table 1 reports patient demographics. Simultaneous CO measurements for all four devices in 17 patients were taken. Two patients were excluded from analysis because of arrhythmias and another was excluded because the arterial pressure waveforms recorded were unusable for the PiCCO device. Table 2 reports CO by device and treatment intervention characteristics. Although mean CO values for PAC, LiDCO, PiCCO and FloTrac were not different (5.6 ± 1.5, 5.4 ± 1.6, 5.4 ± 1.5 and 6.1 ± 1.9 L/min, respectively), mean FloTrac CO values were slightly higher than others, approaching statistical significance between PAC, LiDCO and PiCCO (*P = *0.095, 0.120 and 0.078, respectively).

**Table 1:** Patient demographic dataa

Age (yr)	73 ± 9
Gender (M/F)	11/6
LVEF (%)	52 ± 8
Type of PAC (CO_TD_/CCO)	10/7
Arterial catheter site (femoral/radial)	9/8
Type of operation	Number
CABG	8
AVR	2
MVR	1
AVR + MVR	1
CABG + AVR	3
TAAR	1
CABG + AVR +TAAR	1

^a^Data are presented as means ± SD. LVEF, left ventricular ejection fraction; PAC, pulmonary artery catheter; CO_TD_/CCO, intermittent bolus thermodilution/continuous cardiac output; CABG, coronary artery bypass grafting; AVR/MVR/TVR, aortic/mitral/tricuspid valve repair or replacement; TAAR, thoracic aortic aneurysm repair, *n *= 17.

**Table 2:** Mean cardiac output measurementsa

Baseline CO (L/min), *n *= 17 using both CO_TD _and CCO	
PAC (CO_TD_/CCO)	5.6 ± 1.5
LiDCO Plus	5.4 ± 1.6
PiCCO	5.4 ± 1.5
FloTrac/Vigileo	6.1 ± 1.9
Baseline CO (L/min), *n *= 10 using CO_TD_	
CO_TD _PAC	6.0 ± 1.3
LiDCO Plus	5.9 ± 1.3
PiCCO	6.0 ± 1.3
FloTrac/Vigileo	6.9 ± 1.5
Baseline CO (L/min), *n *= 7 using CCO	
CCO PAC	4.8 ± 1.4
LiDCO Plus	4.5 ± 1.8
PiCCO	4.3 ± 1.5
FloTrac/Vigileo	4.8 ± 1.7
Therapeutic interventions	Number
Vasodilator (any ∆ > 0.1 μg/kg/min in nitroprusside infusion)	34
Vasoconstrictor (any ∆ > 0.01 μg/kg/min in norepinephrine or phenylephrine infusion)	8
Volume challenge (any volume > 250 cc of PRBC, FFP, platelets or 0.9% saline given over < 30 min)	8
Inotrope (any ∆ > 0.01 μg/kg/min in epinephrine or > 1 μg/kg/min in dopamine or dobutamine infusion)	10
Combination of any two or more interventions simultaneously	66

^a^Data are presented as means ± SD, and for characteristics of the events, total number. PAC, pulmonary artery catheter; CO_TD_/CCO, intermittent bolus/continuous cardiac output; ∆, change; PRBC, packed red blood cells; FFP, fresh frozen plasma.

**Figure 1:**
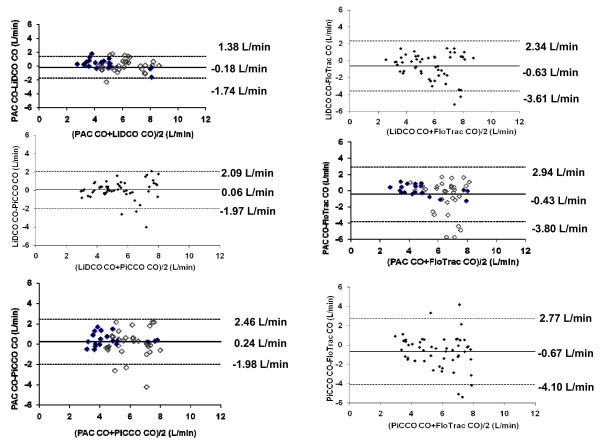
**Bland-Altman analysis of each set of paired devices' cardiac output (CO)**. Solid line, mean difference (bias); dotted lines, limit of agreement (bias ± 1.96 standard deviation (SD)).

The mean CO bias between each paired method was -0.18 (PAC-LiDCO), 0.24 (PAC-PiCCO), -0.43 (PAC-FloTrac), 0.06 (LiDCO-PiCCO), -0.63 (LiDCO-FloTrac) and -0.67 L/min (PiCCO-FloTrac), with limits of agreement (1.96 SD, 95% CI) of ± 1.56, ± 2.22, ± 3.37, ± 2.03, ± 2.97 and ± 3.44 L/min, respectively (Figure 1). The percentage error for each set of paired devices was 29%, 41%, 59%, 39%, 53% and 61%, respectively.

**Figure 2:**
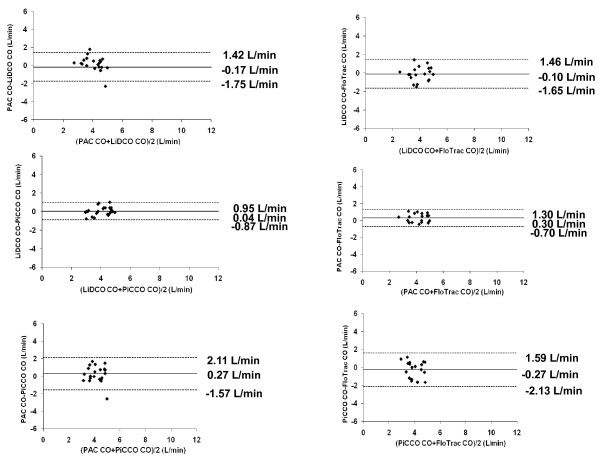
**Bland-Altman analysis of each set of paired devices' cardiac output (CO) ≤5 L/min**. Solid line, mean difference (bias); dotted lines, limits of agreement (bias ± 1.96 SD).

Since CO accuracy may be clinically more important at low CO values, we analyzed the agreement among estimates of CO for mean values ≦5 L/min. For CO values ≦5 L/min, bias and limits of agreement were -0.17 ± 1.58 (PAC-LiDCO), 0.27 ± 1.84 (PAC-PiCCO), 0.30 ± 1.00 (PAC-FloTrac), 0.04 ± 0.91(LiDCO-PiCCO), -0.10 ± 1.56 (LiDCO-FloTrac) and -0.27 ± 1.86 L/min (PiCCO-FloTrac) (Figure 2).

**Figure 3:**
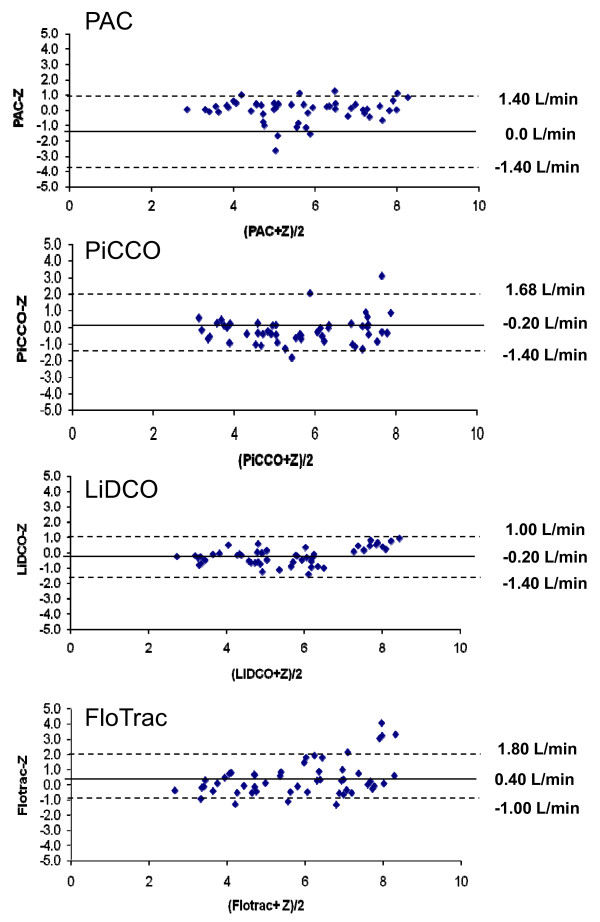
**Bland-Altman analysis of each device against the mean of all devices across all patients, wherein pulmonary arterial catheter (PAC) thermodilution CO (COtd) and continuous CO (CCO) are pooled to be one variable (*Z*-statistic)**. Solid line, mean difference (bias); dotted line, limits of agreement (bias ± 1.96 SD).

The mean CO bias between each device and the pooled group CO values, noting individual device variance from the group mean, was -0.2 (LiDCO), 0.4 (FloTrac), -0.2 (PiCCO) and 0.0 L/min (PAC), with limits of agreement (1.96 SD, 95% CI) of ± 1.2 ± 2.4 ± 1.6 and ± 1.4, respectively (Figure 3).

### PAC COtd vs. CCO as reference points

**Figure 4:**
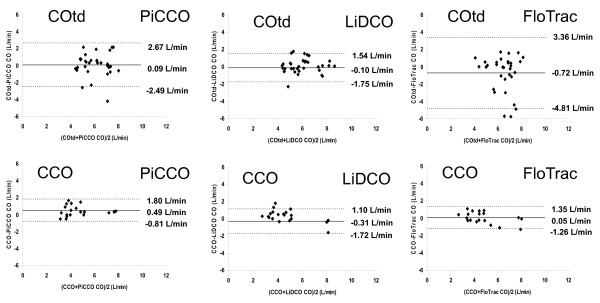
**Bland-Altman analysis of subgroups of patients with either thermodilution cardiac output (CO_TD_) or CCO PAC (*Z*-statistic)**. Solid line, mean difference (bias); dotted lines, limits of agreement (bias ± 1.96 SD).

The bias and limits of agreement for each paired method in subgroup analyses of patients with either CO_TD _or CCO PAC are shown in Figure 4. The bias and limits of agreement for LiDCO with CCO (-0.31 ± 1.41 L/min), PiCCO with CCO (0.49 ± 1.30 L/min) and FloTrac with CCO (0.05 ± 1.30 L/min) were different from that of the three devices with CO_TD _PAC (-0.10 ± 1.64, 0.09 ± 2.58 and -0.72 ± 4.09 L/min, respectively).

**Figure 5:**
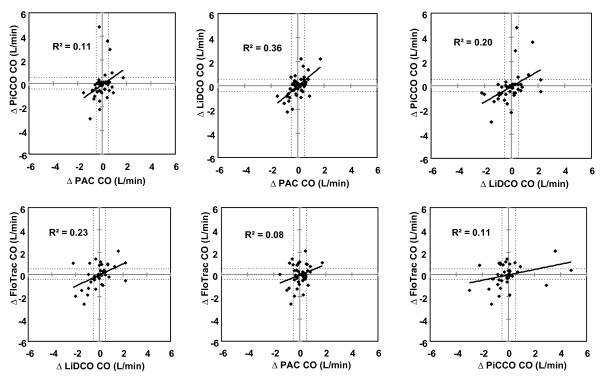
**Pearson product-moment analysis of change in cardiac output (∆CO; in L/min) by each set of paired devices**. Dotted lines, CO of ± 0.5 L/min.

The directional changes between any two paired CO measurements from before and after each intervention displayed 74% (PAC-LiDCO), 72% (PAC-PiCCO), 59% (PAC-FloTrac), 70% (LiDCO-PiCCO), 71% (LiDCO-FloTrac) and 63% (PiCCO-FloTrac) concordance but poor correlation (*r*^2 ^= 0.36, *P <*0.0001; *r*^2 ^= 0.11, *P *= 0.025; *r*^2 ^= 0.08, *P *= 0.079; *r*^2 ^= 0.20, *P *= 0.002; *r*^2 ^= 0.23, *P *= 0.001; and *r*^2 ^= 0.11, *P *= 0.033, respectively) (Figure 5).

## Discussion

DO_2_-targeted resuscitation protocols reduce both length of stay and infectious complications in high-risk surgical patients [27,28]. Several minimally invasive monitoring devices have been used to realize these benefits. Our study demonstrates that the three commercially available CO monitoring devices report similar mean CO values, but dynamic trends among these devices over clinically relevant CO changes are not consistent. Thus, in the presence of no contradictory findings, one must use monitors specifically used in a proven effective treatment protocol to ensure the utility of that treatment. Within this context, PAC, LiDCO plus™ and FloTrac postoptimization protocols have been shown to improve patient-centered outcome [27,29,30]. Surprisingly, no comparable PAC data-specific clinical trials have been reported. We are unable to comment on the ability of FloTrac™- or PiCCO plus™-guided therapy to improve outcome because they have not been studied in this context. However, on the basis of our analysis of 55 quadruple measures and the three recent clinical trials [18-21,31], it is doubtful that their performance, using the present proprietary iterations, will be interchangeable with PAC or result in any better outcomes than were observed using the LiDCO plus™ CO estimates to target DO_2 _levels.

This clinical study is unique for two specific reasons. First, we studied three commercially available pulse contour-pulse power analysis devices that report continuous CO measures and compared them to each other and to two types of PAC CO estimates: COtd or CCO. Since none of these devices is a "gold standard," the three pulse contour devices were compared to each other and to the PAC as equal devices. Our comparisons show that LiDCO plus™ and PAC have greater agreement with each other than do either PiCCO plus™ or FloTrac™ with PAC. Furthermore, the limits of agreement between LiDCO plus™ and PAC are within the boundaries of the Critchey-Critchey criteria [25], whereas those of PiCCO plus™ or FloTrac and PAC exceed those criteria. This close correlation also agrees with our previous data during open heart surgery, wherein we documented that the LiDCO plus™ estimates of stroke volume accurately trend actual left ventricular stroke volume measures during rapid and dynamic changes in CO when aortic flow was accurately measured in humans using an electromagnetic flow probe placed around the ascending aorta [32]. These levels of agreement difference persist when all devices are compared to a mean pooled CO value of the group as opposed to each other separately (Figure 3). Second, we studied three separate types of resuscitation interventions (volume loading, vasoactive drug use and inotropic agent use) which reflect clinically relevant scenarios. To date, all published validation studies cited above examined only the ability of these devices to track cardiac output changes in response to volume loading when vasoactive drug therapy was held constant. Although changes in CO in response to volume loading are very important to document, the impact of other vasoactive therapies are equally important, commonly seen in the clinical setting and potentially confounding to the accuracy of pulse pressure-derived estimates of CO.

In support of our findings, recent studies with FloTrac™ showed limited accuracy compared to PAC [18,19,31]. Mayer *et al*. [31] showed in intraoperative cardiac surgery patients that FloTrac™ displayed an overall percentage error of 46% compared to paired COtd values. Potentially, these previous studies unfairly studied FloTrac™ by using profound vasomotor paralysis and flow labile states, a clinical limitation specifically cautioned by the manufacturer. Our FloTrac™ device was equipped with the second-generation software modified to be more accurate in labile states. However, Compton *et al*. [33] reported continued poor limits of agreement between this second-generation FloTrac™ algorithm and PiCCO plus™ thermodilution CO measures. Thus, our FloTrac™-PAC data agree with their findings. FloTrac™ has subsequently developed a third-generation software algorithm that we did not use. We do not know if this newer iteration will improve FloTrac™ accuracy, since that modification allowed FloTrac™ CO estimates to remain accurate during decoupling states, such as sepsis, which were conditions not present in our cohort. Conversely, PiCCO plus™ calibration appears to remain accurate within 6 h of calibration even when vascular tone has been changed [34].

We had nearly equal numbers of patients studied with CO_TD _and CCO PAC. This allowed us to compare these measures with pulse contour analysis. Since both CO_TD _and CCO are clinically acceptable as part of standard of care in the ICU, this distribution of patients makes our data more robust as a reference for standard ICU care. Regrettably, both FloTrac™ and LiDCO Plus™ CO values had poor bias and precision with PAC-derived CO values for both COtd and CCO. These findings are also consistent with the findings of others [18,19,35-37]. Since we did not compare COtd to CCO in the same patient because of the observational nature of our study, we cannot comment on the potential bias between COtd and CCO. However, independently of which PAC method was used for these comparisons, neither gives actual instantaneous measures of CO. COtd measures require the averaging of three to five separate measures taken over a 5-min interval. If cardiac output is systematically changing during this interval (that is, either increasing or decreasing from the start to the end of the series of thermodilution measures), the calculated CO value may not reflect instantaneous CO values taken at the same time. Similarly, CCO uses a moving average algorithm that examines thermal dilution of 3 min, making it highly insensitive to rapid changes in CO. However, in our study, we were concerned only with defining the data collection times as those following specific therapeutic interventions when hemodynamic measures, including heart rate, CO and mean arterial pressure, were constant. Although such statements of stability are relative considering the unstable nature of the postoperative cardiac surgery patient, for the purposes of CO measures they were stable over the 5 min of data collection.

Since absolute CO measures become increasingly more important at low CO values [38,39], we assessed agreement among our monitoring devices by *post hoc *analysis of all measured CO values ≤5 L/min. We found that the degree of bias decreased slightly relative to the complete CO data set, although the degree of variability among the devices remained (Figure 2). Accordingly, LiDCO Plus™, PiCCO Plus™ and FloTrac™ cannot be assumed to be interchangeable with PAC devices in the assessment of low CO values. Again, which device, if any, reports the most accurate value and trend during low flow states is not known on the basis of our study. Furthermore, most of the variance between LiDCO™ and FloTrac™ with PAC-derived CO measures came from the CO_TD _values, and then when these cardiac output values were > 5 L/min. This finding is the opposite of what Opdam *et al*. found [18]. Potentially, averaging CO measurements over 20 s improved agreement between the devices and CCO as opposed to those and CO_TD _PAC. This difference between CCO and COtd may reflect the clinical decision bias by which patients with intrinsically lower CO get CCO devices (4.8 ± 1.4 l/min), whereas those with high CO get CO_TD _devices (6.0 ± 1.3 l/min).

One major potential benefit of using CCO monitoring is to note directional changes in flow. By Pearson product-moment analysis, we found poor correlation between each device pair, with the best correlation between LiDCO Plus™ and FloTrac™. PiCCO Plus™ Pearson product-moment analysis accuracy was intermediate between LiDCO™ and FloTrac™.

That these devices differed in their paired performances is not surprising. They all use different aspects of the arterial pulse and rely on different assumptions in their CO estimations. Most of our patient cohort was being administered varying levels of vasoactive medications that must alter their vasomotor tone at baseline and over time. Since LiDCO Plus™ and FloTrac™ use similar aspects of the arterial pulse to calculate CO, this may explain their better concordance by Pearson product-moment analysis. Also, volume challenge in preload-responsive patients increases CO by > 10%-15% [33,40]. We used this threshold CO value as a minimal CO change and still observed poor agreement between devices.

### Study limitations

First, we report on a small patient cohort, limiting subgroup analysis and potentially showing differences when a larger number of patients would show similarity. Not all patients received all therapies, since our study was observational. Still, this limitation reflects real-life conditions. Yet, patients are treated individually, not as group means, thus these data are relevant to clinical decision making. Second, we did not use the PiCCO™ or LiDCO™ device-specific calibration methods. However, our common baseline external calibration method is approved by both manufacturers as an acceptable method. Since our goal was to ascertain the dynamic accuracy of these devices, we reasoned that starting from a common CO value using an external calibration method would maximize potential CO agreements between devices. If anything, separate PiCCO™ and LiDCO™ calibrations would produce more, not less, CO variance than we report. Third, we compared not only mean CO values but also their changes and Pearson product-moment analysis as recommended by Squara *et al*. [21]. They also recommended assessment of dynamic real-time trends as a fourth method of analysis. We did not use this fourth method of comparison, because COtd did not lend itself to it. Finally, not all of our patients had femoral arterial catheters, which might have affected the result of PiCCO™ CO estimates, as large peripheral arteries are their preferred sites. However, the femoral (central arterial) site requirement is such that the thermal calibration signal can pass the sensing thermistor not for subsequent CO estimates. The manufacturer allows for radial site insertion with external calibration. Furthermore, we saw no systematic differences in agreement from femoral and radial site PiCCO CO measures. Thus, the PiCCO data reflect the accurate values.

## Conclusions

LiDCO Plus™, PiCCO™, FloTrac™ and PAC did not show similar CO trending results, although all produced similar pooled steady-state CO values. Furthermore, if clinical trials of resuscitation based on CO values show efficacy when using one of these devices, it is not clear whether performing the identical trial with another CO monitoring device will also show similar benefit. Thus, until the agreement among minimally invasive CO measuring devices improves, each device needs to have its own clinical efficacy validated.

## Key messages

• Since the PAC-derived estimates of cardiac output by the thermodilution technique are not the gold standard for estimating cardiac output at the bedside, all available measures of cardiac output need to be compared to each other rather than to a PAC reference.

• Different commercially available arterial pressure-derived estimates of cardiac output give differing degrees of error relative to each other.

• The cardiac output error among devices is low for cardiac output values < 5 L/min.

• Studies documenting clinical benefit using catheter-derived estimates of cardiac output to drive resuscitation algorithms using one monitoring device cannot be extrapolated to similar utility by using another cardiac output monitoring device.

## Abbreviations

CCO: cardiac output by continuous technique; CO: cardiac output; COtd: cardiac output by thermodilution technique; DO_2_: oxygen delivery; ICU: intensive care unit; PAC: pulmonary artery catheter.

## Competing interests

MRP is a member of the medical advisory boards for and has received honoraria for lectures from both LiDCO Ltd and Edwards LifeSciences, Inc, and has stock options with LiDCO Ltd. All other authors declare that they have no competing interests.

## Authors' contributions

MH helped design the study, recruited the patients, collected the data, analyzed the initial data and wrote the first draft of the manuscript. HKK helped analyze the data and edited the later versions of the manuscript. DS helped collect and store the data and performed the preliminary statistical analysis. MRP helped design the study, got Institutional Review Board approval, analyzed the data and wrote all versions of the manuscript.

## Acknowledgements

The authors thank the Cardiothoracic Intensive Care Unit nursing staff at Presbyterian University Hospital, University of Pittsburgh Medical Center, for their support in conducting the study. Also, we appreciate both Edwards LifeSciences and LiDCO companies for providing us with the devices, supplies and training for the study. This work was supported in part by National Institutes of Health grants HL67181 and HL073198.
